# Angiogenesis in human brain tumors: screening of drug response through a patient-specific cell platform for personalized therapy

**DOI:** 10.1038/s41598-018-27116-7

**Published:** 2018-06-08

**Authors:** Laura Guarnaccia, Stefania Elena Navone, Elena Trombetta, Chiara Cordiglieri, Alessandro Cherubini, Francesco Maria Crisà, Paolo Rampini, Monica Miozzo, Laura Fontana, Manuela Caroli, Marco Locatelli, Laura Riboni, Rolando Campanella, Giovanni Marfia

**Affiliations:** 1 Laboratory of Experimental Neurosurgery and Cell Therapy, Neurosurgery Unit, Fondazione IRCCS Ca’ Granda Ospedale Maggiore Policlinico, University of Milan, Milan, Italy; 2 Flow Cytometry Service, Laboratory of Clinical Chemistry and Microbiology, Fondazione IRCCS Ca’ Granda Ospedale Maggiore Policlinico Milan, Milan, Italy; 3 Istituto di Genetica Molecolare “Romeo ed Enrica Invernizzi”, Milan, Italy; 4 Cell Factory, Unit of Cell Therapy and Cryobiology, Fondazione IRCCS Ca’ Granda Ospedale Maggiore Policlinico, Milan, Italy; 5 Division of Pathology, Department of Pathophysiology and Transplantation, University of Milan, Fondazione IRCCs Ca’ Granda Ospedale Maggiore Policlinico, Milan, Italy; 6 Department of Medical Biotechnology and Translational Medicine, LITA-Segrate, University of Milan, Milan, Italy

## Abstract

Gliomas are the most common brain tumors, with diverse biological behaviour. Glioblastoma (GBM), the most aggressive and with the worst prognosis, is characterized by an intense and aberrant angiogenesis, which distinguishes it from low-grade gliomas (LGGs) and benign expansive lesions, as meningiomas (MNGs). With increasing evidence for the importance of vascularization in tumor biology, we focused on the isolation and characterization of endothelial cells (ECs) from primary GBMs, LGGs and MNGs. Gene expression analysis by Real-Time PCR, immunofluorescence and flow cytometry analysis, tube-like structures formation and vascular permeability assays were performed. Our results showed a higher efficiency of ECs to form a complex vascular architecture, as well as a greater impairment of a brain blood barrier model, and an overexpression of pro-angiogenic mediators in GBM than in LGG and MNG. Furthermore, administration of temozolomide, bevacizumab, and sunitinib triggered a different proliferative, apoptotic and angiogenic response, in a dose and time-dependent manner. An increased resistance to temozolomide was observed in T98G cells co-cultured in GBM-EC conditioned media. Therefore, we developed a novel platform to reproduce tumor vascularization as “disease in a dish”, which allows us to perform screening of sensitivity/resistance to drugs, in order to optimize targeted approaches to GBM therapy.

## Introduction

Central Nervous System (CNS) tumors are among the most complex human cancers, with a prognosis closely related to histological classification, location, genetic background and specific microenvironment of the tumor. Brain tumors present anatomically similar features, but they differ significantly for morphology, aetiology, site of origin, molecular biology and clinical progression^1^. In adults, about 50% of all CNS tumors are malignant, whereas in paediatric patients, more than 75%^2^. The ability of tumor mass to grow, progress and infiltrate into surrounding tissue, compromising cognitive and motor skills, is due to the intense and aberrant angiogenesis, responsible for the formation of new blood vessels, which support tumor development, providing nutrition, oxygen and energy^3^, in a mechanism well known as neovascularization. Angiogenesis is regulated by a number of both stimulating and inhibiting angiogenic factors acting on endothelial cells (ECs)^4^. Often, concentration of pro-angiogenic mediators increase progressively with tumor grade and is linked with the clinical outcome^5^. In this complex process of neovascularization, tumor ECs are the protagonists, and their cellular and molecular properties, specific of tumor grade, affect their functionality and, consequently, tumor response to therapies. Microvascular proliferation, indeed, is a key feature of glioma grading: it is quite absent in low grade gliomas (LGGs, WHO II) and observed in malignant high-grade gliomas, HGGs (WHO, grades III, and IV), the tumor with the worst prognosis. On the contrary, although meningiomas (MNGs, WHO grade I) represent tumors with a very high degree of vascularization, they show non-aggressive biology, resulting in the most favourable long-term survival (median, 12–15 years)^6^. The most common and malignant human intracranial cancer is glioblastoma (GBM), classified as WHO grade IV, with a complex biology characterized by uncontrolled proliferation, diffuse infiltration, hypervascularization and resistance to therapies. Standard first line management for newly diagnosed GBM involves a multi-modality approach, including surgical resection, followed by radiation with concurrent and adjuvant Temozolomide (TMZ). Despite countless experimental researches for new therapeutic strategies and promising clinical trials, the prognosis remains extremely poor, with a mean survival of less than 14 months^7,8^. Bevacizumab (BEV), a humanized monoclonal antibody against vascular endothelial growth factor (VEGF), has increasingly been used in the treatment of recurrent GBM, due to promising trial results demonstrating improved response rates. Unfortunately, BEV effects are only transient and GBM recurrence is not avoided^9^. Interestingly, sunitinib (SUN), a multitargeted antiangiogenic tyrosine kinase inhibitor (TKI) has attracted the attention of the scientific community for its ability to inhibit angiogenesis in different tumors^10,11^, proving to be a potential candidate to counteract neovascularization in GBM. The ability of microvascular ECs to respond dynamically to pathology-related microenvironment changes is particularly apparent in tumor-growth-associated angiogenesis. The heterogeneity of brain tumors, as well as the need to improve patient response to therapies, in terms of progression-free survival and quality of life, brings to mind the idea of developing patient-specific personalized therapies, based on cellular response to treatments. In this regard, the possibility to develop a primary EC characterization platform, biologically as close as possible to the *in situ* situation, as “disease in a dish”, greatly valorises brain tumor angiogenesis investigation, improving pharmacologic screening and, in turn, target therapies.

## Results

### Phenotypic analysis revealed a higher pro-angiogenic expression pattern in GBM than in LGG and MNG

**Figure 1:**
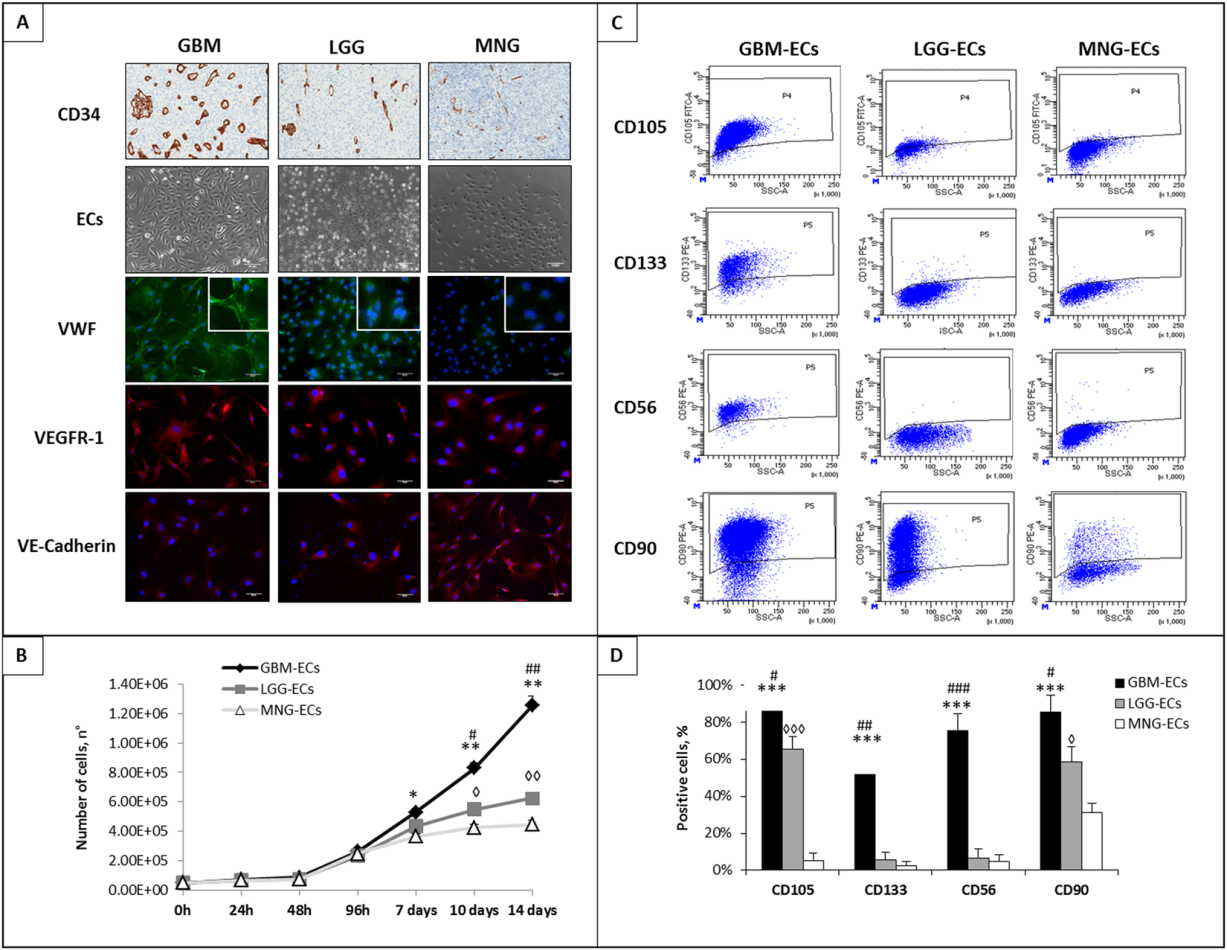
Isolation, expansion and characterization of primary GBM, LGG and MNG-derived ECs. (**A**) Immunohistochemical staining for CD34 in GBM, LGG and MNG sections revealed an higher expression in GBM than in LGG and MNG. After tumor sample processing, GBM-ECs, LGG-ECs and MNG-ECs were isolated and expanded. As shown, ECs were able to form a “cobblestone” monolayer (inverted phase-contrast microscopy). Immunofluorescence labelling for VWF (green fluorescent labelling), VEGFR-1(red fluorescent labelling) and VE-Cadherin(red fluorescent labelling) was performed on cultured ECs. The white box contains an enlargement of the image. Scale bar 50 µm. (**B**) Estimation of proliferation rate of GBM-ECs, LGG-ECs, MNG-ECs for 14 days after seeding. (**C**) Representative cytofluorimetric dot plots of GBM-ECs, LGG-ECs and MNG-ECs stained with FITC-conjugated anti-human CD105 and PE-conjugated anti-human CD133, anti-human CD56, anti-human CD90. Analysis were performed after doublets and dead cells exclusion. (**D**) Quantification of the positive cell population in percentage. Data are the means of ±SD of three independent experiments. *GMB vs MNG, ^#^GBM vs LGG, ^◊^LGG vs MNG. *^#◊^P < 0.05, **P < 0.01, ***^, ###, ◊◊◊^P < 0.001

Immunoistochemical labelling proved the higher CD34 expression pattern in GBM sections compared to those of LGG and MNG. Quantification of integrated density reported an average value for field of 25,7 ± 3,65 (AU) for GMBs, 7,82 ± 1,03 for LGGs and 3,96 ± 1,26 for MNGs (Fig. 1A). Primary ECs isolated from GBMs, LGGs and MNGs were cultured and expanded in Endothelial Proliferation medium (EndoPM), growing as a typical cobblestone monolayer (Fig. 1A). Immunofluorescence analysis revealed positivity for the endothelial markers von Willebrand factor (VWF), vascular endothelial growth factor receptror-1 (VEGFR-1) and VE-Cadherin, thus confirming their endothelial phenotype (Fig. 1A). In particular, GBM-ECs were able to form VWF fibers, confirming the higher activation level of ECs derived from GBMs, compared to those derived from LGGs and MNGs. As notable, fluorescence intensity is considerably greater in GBM-ECs compared to LGG-ECs and MNG-ECs, not only regarding VWF but also VEGFR-1, suggesting an overexpression of endothelial functional markers, essential for angiogenesis promotion. On the contrary, a lower intensity in GBM-ECs compared to LGG-ECs and MNGs was observed for VE-Cadherin labelling. Notably, no positive labelling for GFAP, SMA and CD68 was observed (Supplementary Table 1).

### GBM-ECs grow with an exponential rate, unlike LGG- and MNG-ECs

In order to estimate the proliferation rate of ECs, a growth curve of 14 days was performed. In the first steps of cell cultures, GBM-, LGG- and MNG-ECs showed the same proliferation rate. Up to 96 h after seeding in fact, ECs grew with the same trend, reaching a cell number of about 5-fold higher than at start time. However, from 48 h to 14 days, GBM-ECs grew with an exponential proliferation rate, being in number 2- and 3-fold higher than LGG-ECs and MNG-ECs, respectively. On the contrary, LGG-ECs and MNG-ECs showed a rather exponential proliferation rate from 48 h to 7 days, after which they slowed growth, reaching a plateau stage (Fig. 1B).

### FACS analysis showed a different expression profile in brain tumor ECs

FACS analysis revealed interesting different and specific expression pattern, closely related to tumor malignancy and neovascularization. CD105 was overexpressed in GBM-ECs compared to LGG-ECs and MNG-ECs (Fig. 1C). In the ECs populations analysed, about 90 ± 5% of GBM-ECs were positive for CD105, whereas 60 ± 7% and 5 ± 4% of LGG-ECs and MNG-ECs were positive, respectively. GBM-ECs positivity of 55 ± 12% for CD133, compared to LGGs and MNGs where CD133-positive ECs were only about 6 ± 4% and 3 ± 2% respectively. CD56 and CD90 showed the same trend. About 75 ± 9%, 6 ± 5% and 4 ± 3% of ECs derived from GBMs, LGGs and MNGs were positive to CD56, whereas 96 ± 9%, 68 ± 8% and 26 ± 5% were positive to CD90, respectively (Fig. 1D).

### GBM-ECs, LGG-ECs and MNG -ECs showed a different ability to form tube-like structures

**Figure 2:**
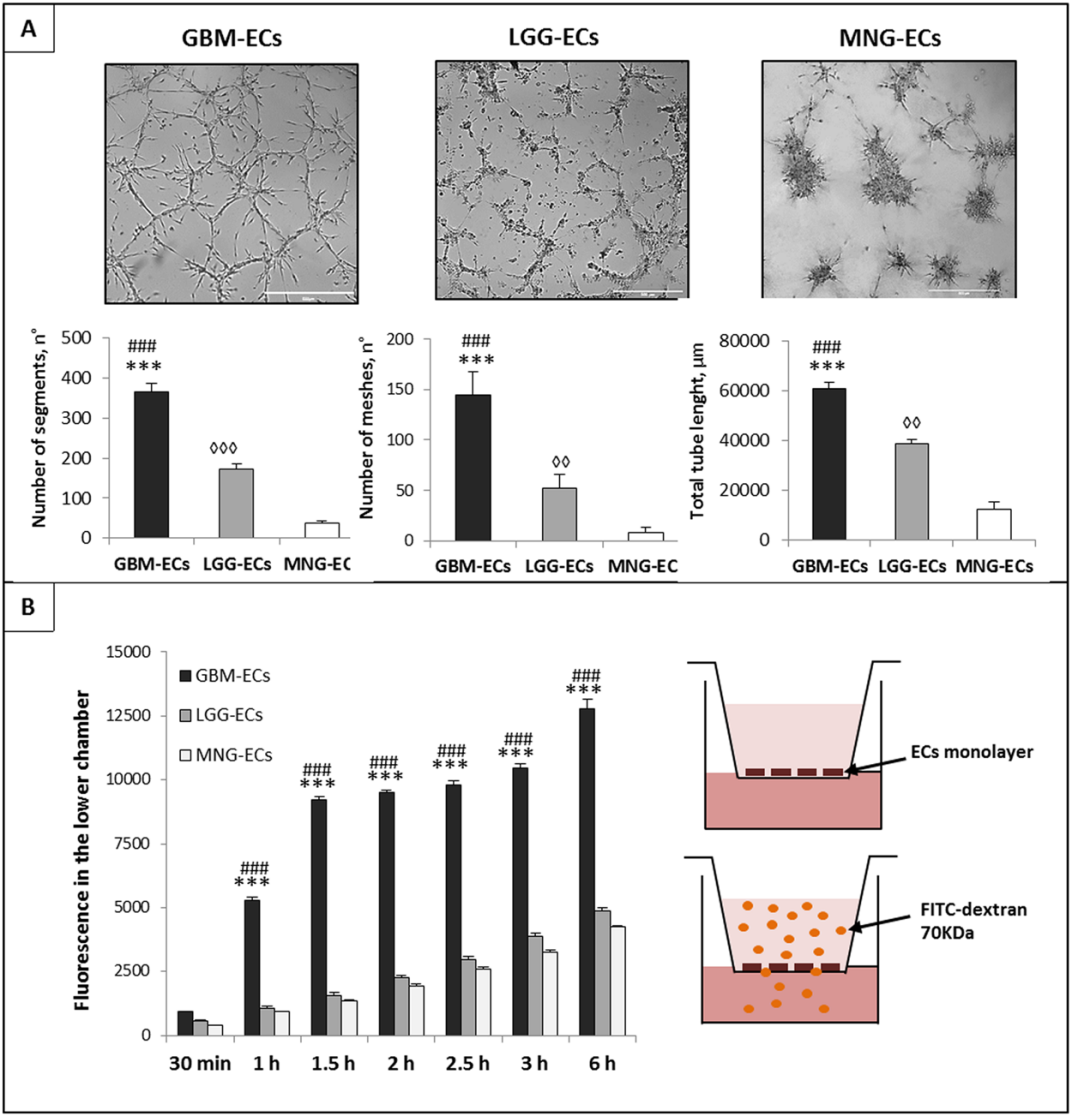
Tube-like structure formation and vascular permeability assays. (**A**) Representative images of tube-like structures formation assay performed on GBM-ECs, LGG-ECs and MNG-ECs seeded on Matrigel. Numbers of segment, meshes and total tube length were measured using Angiogenesis Analyzer plugin in ImageJ. Data are the mean ± SD of at least three experiments in triplicate. Scale bar, 500 µm. (**B**) Vascular permeability assay performed administrating FITC-70 kDa on GBM-ECs, LGG-ECs and MNG-ECs confluent monolayer cultured in Transwell insert. Data are the mean ± SD of three experiments in triplicate. *GBM vs MNG, ^#^GBM vs LGG, ^◊^LGG vs MNG. *P < 0.05, **^, ◊◊^P < 0.01, ***^, ###, ◊◊◊^P < 0.001.

The analysis of tube-like structures formation assay (Fig. 2A), revealed that GBM-ECs were able to form a complex and branched architecture. After 24 h, GBM-ECs were able to form junctions, cords and meshes, showing the ability to create an intense vasculature. On the contrary, LGG-ECs and MNG-ECs failed to form tube-like structures, lacking of an organized network. In particular, GBM-ECs succeeded to form a number of segment 2- and 4-fold higher than LGG- and MNG-ECs respectively, as well a number of meshes about 3- and 6- fold higher. Regarding total tube length, we observed an increase of about 1.5- and 3-fold in GBM-ECs compared to LGG- and MNG-ECs.

### GBM-ECs present an increased vascular permeability compared to LGG-ECs and MNG-ECs

Vascular permeability assay was performed in order to evaluate the different permeability rate of ECs derived from different brain tumors and investigate the functionality of BBB. The histogram in Fig. 2B shows the amount of FITC-70 kDa dextran that succeeded to cross the cellular barrier formed by GBM-EC, LGG-EC and MNG-EC monolayer. As shown, there was a notable time-dependent trend, so the concentration of dextran detectable in the lower chamber increased over time. Notably, the amount of FITC-70 kDa dextran that passed across GBM-ECs barrier was significantly higher compared to LGG-ECs and MNG-ECs samples, indicating that GBM-ECs form a weak and defenestrated barrier.

### Brain tumor derived ECs showed significant differences in mRNA expression of angiogenic mediators

**Figure 3:**
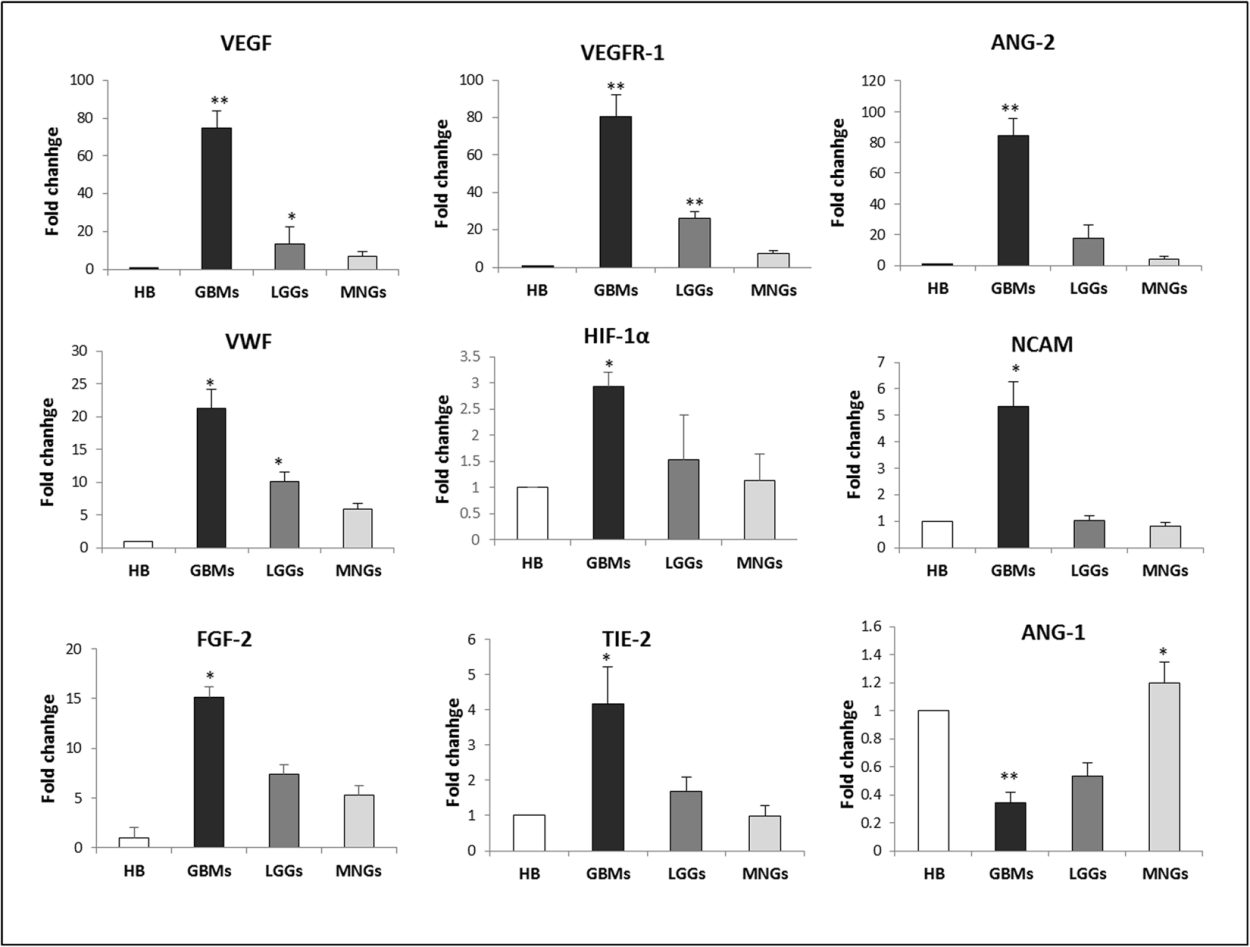
Gene expression profile of GBM, LGG and MNG tumor tissues. qRT-PCR performed on tumor samples homogenates. Data were calculated using the 2^−^ΔΔCt method, normalized to 18S expression, and results are presented as a fold change relative to healthy brain (HB). Primer sequences are listed in the Supplementary Table 2. Data are the mean of ±SD of three independent experiments. *GBM, LGG, MNG vs HB *P < 0.05, **P < 0.01.

In order to investigate the complex microenvironment of brain tumors, qRT-PCR was performed on the entire tumor sample homogenates of GBMs, LGGs and MNGs (Fig. 3). Results were compared to those obtained from healthy brain (HB), used as control. Our data reported that vascular endothelial growth factor (VEGF) mRNA was up 70-, 15- and 7-fold overexpressed in GBM, LGG and MNG, respectively, compared with HB. Similar results were observed in mRNA expression of both VEGFR-1 and angiopoietin-2 (Ang-2), whose levels were about 80-, 20- and 8-fold higher in GBM, LGG, and MNG, respectively, compared with HB. Notably, VWF, hypoxia inducible factor 1 (HIF-1), neural cell adhesion molecule (NCAM), fibroblastic growth factor-2 (FGF-2), and angiopoietin receptor Tie-2 mRNA levels followed a similar trend, showing a significant up-regulation in GBM, compared to LGG, MNG, and HB. On the contrary, mRNA expression of Ang-1 was significantly lower in GBM and LGG, whereas was quite similar in MNG.

### Drug treatment effects on GBM-ECs viability

**Figure 4:**
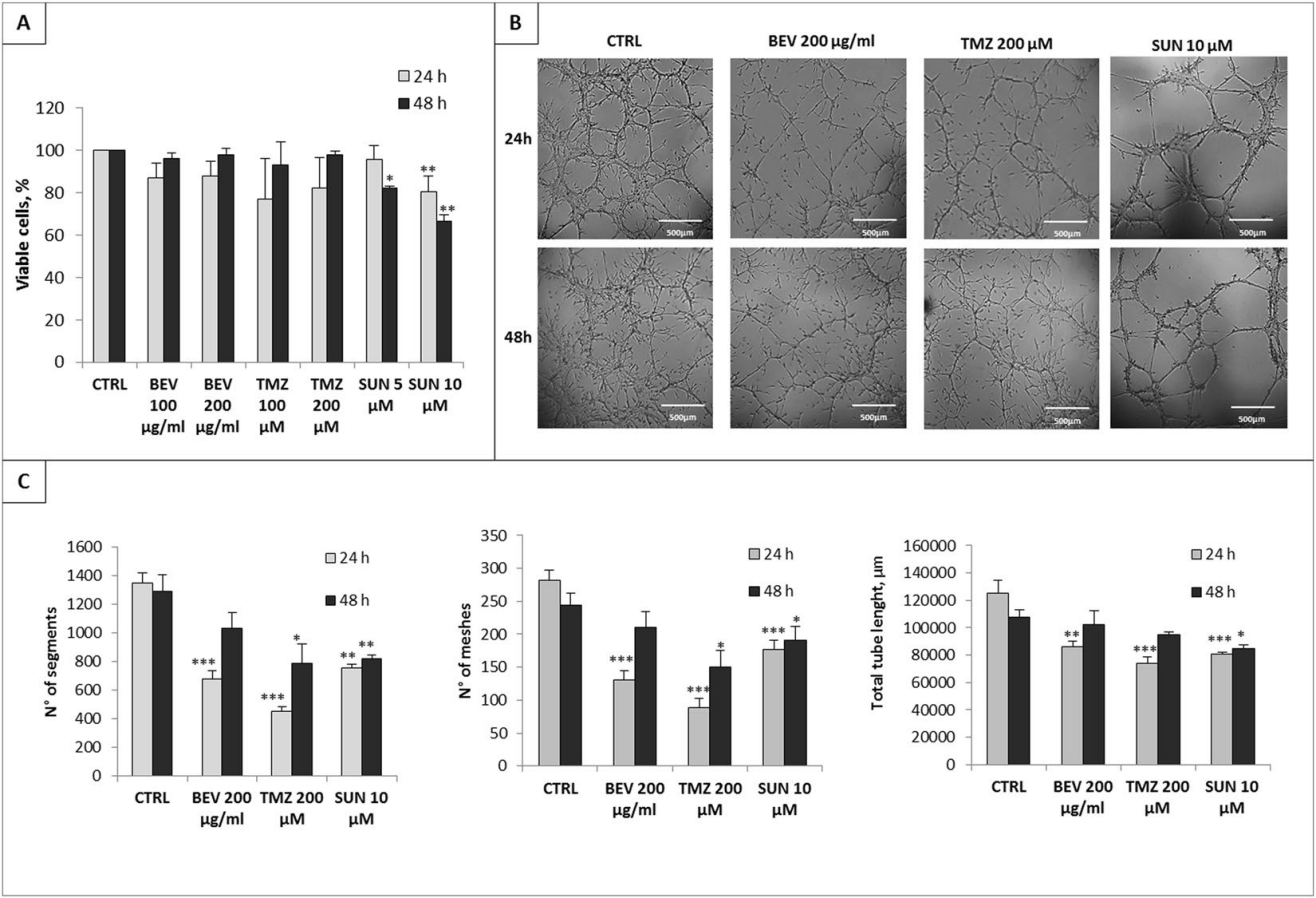
Tube-like structures formation and cell viability on GBM-ECs after drugs administration. (**A**) MTT assay on GBM-ECs treated with BEV 100 µg/mL and 200 µg/mL, TMZ 100 µM and 200 µM, SUN 5 µM and 10 µM after 24 h and 48 h. Data are presented as a percentage of viable cell relative to untreated control (CTRL). (**B**) Representative images of tube-like structures formation assay performed on GBM-ECs cultured in basal condition or in combination with BEV 200 µg/ml, TMZ 200 µM and SUN 10 µM for 24 h and 48 h. (**C**) Numbers of segment, meshes and total tube length were measured using Angiogenesis Analyzer plugin in ImageJ. Data are the mean ± SD of at least three experiments in triplicate. Scale bar, 500 µm. *BEV, TMZ, SUN vs CTRL. *P < 0.05, **P < 0.01, ***P < 0.001.

In order to test drug effect on GBM-EC viability, MTT assay was performed 24 h and 48 h after treatment. Data revealed that BEV and TMZ were able to decrease cells viability after 24 h at 87% and 81% for BEV 100 and 200 µg/mL and 82% and 77% for TMZ, 100 and 200 µM, respectively. Notably, at 48 h their effect disappeared, suggesting that the initial positive effect of pharmacological inhibition did not last over time. Indeed, following 48 h of treatment the viability was quite similar to untreated control. On the contrary, SUN at 5 µM had almost no effect at 24 h, when viable cells were about 95%, whereas at 10 µM viability decreased at about 80%. Interestingly, after 48 h, SUN effect was enhanced, and viability was about 82% and 66% at 5 µM and 10 µM, respectively (Fig. 4A).

### Tube-formation ability of GBM-ECs is differently affected by drugs administration

BEV, TMZ and SUN proved the ability to inhibit significantly tube-like structures formation after 24 h of treatment. Indeed, as shown in Fig. 4B,C, at 24 h, number of segments and meshes was approximately halved after treatment with BEV 200 µg/ml and TMZ 200 µM and also total tube length was significantly decreased. It is noteworthy that these data were not confirmed at 48 h. Indeed, after prolonged treatment, ECs seem to recover their ability to form tube-like structures, mitigating or even abolishing BEV and TMZ inhibition. Interestingly, GBM-ECs treated with SUN showed a different behaviour. The effect of SUN, in fact, at 24 h was lower compared to that of BEV and TMZ, but it was constant over time, both in terms of number of segments, meshes and total tube length.

### Gene expression performed with qRT-PCR suggest a different response of GBM-ECs to drugs

**Figure 5:**
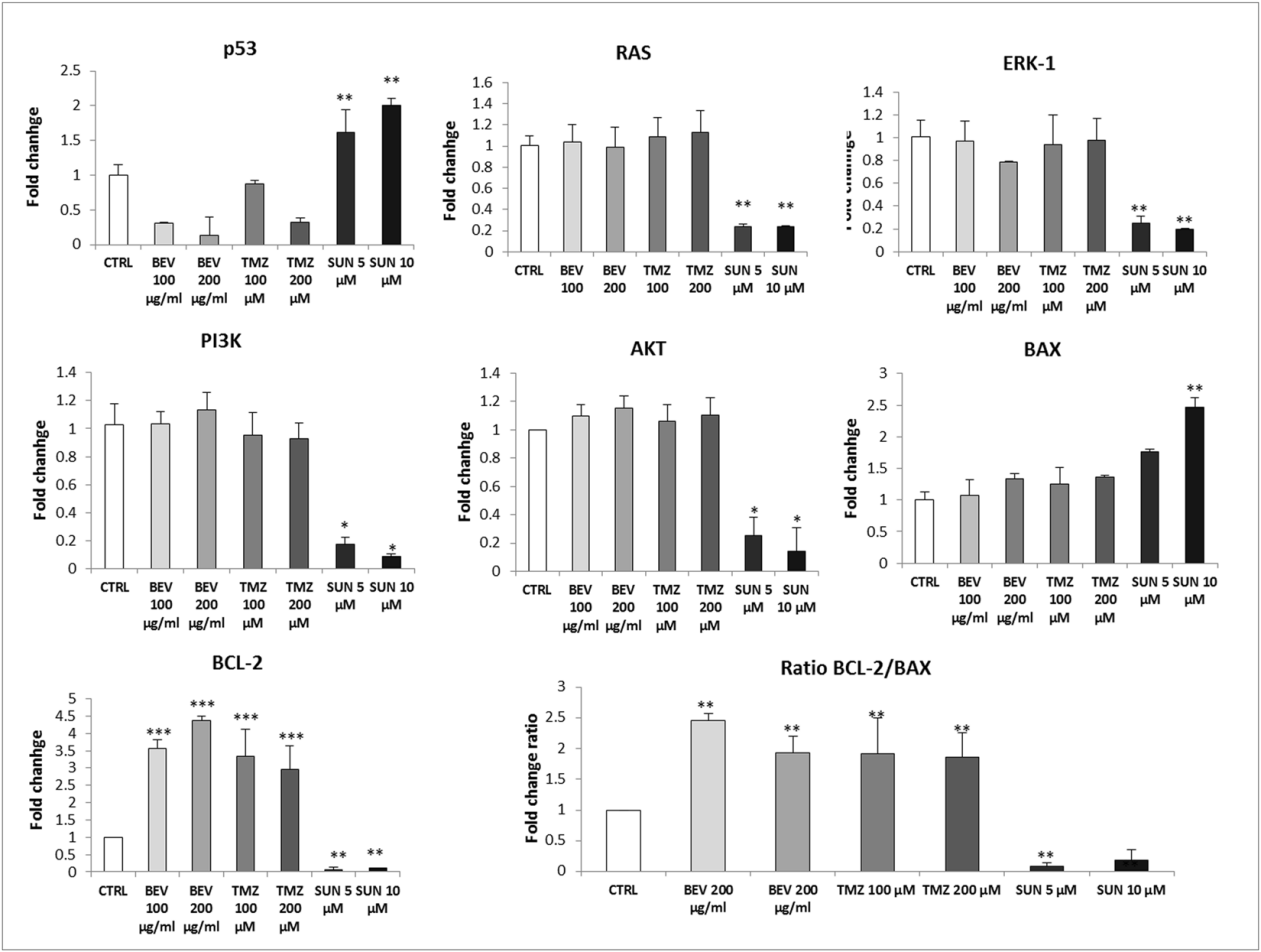
Gene expression profile of proliferative and apoptotic markers on GBM-ECs after drug administration. qRT-PCR was performed on GBM-ECs cultured in basal condition (CTRL) or in combination with BEV 100 µg/mL and 200 µg/mL, TMZ 100 µM and 200 µM, SUN 5 µM and 10 µM for 48 h. Data were calculated using the 2^−^ΔΔCt method, normalized to 18S expression, and results are presented as a fold change relative to untreated control (CTRL). Primer sequences are listed in the Supplementary Table 2. Data are the mean of ±SD of three independent experiments. *BEV, TMZ, SUN vs CTRL. *P < 0.05, **P < 0.01, ***P < 0.001.

**Figure 6:**
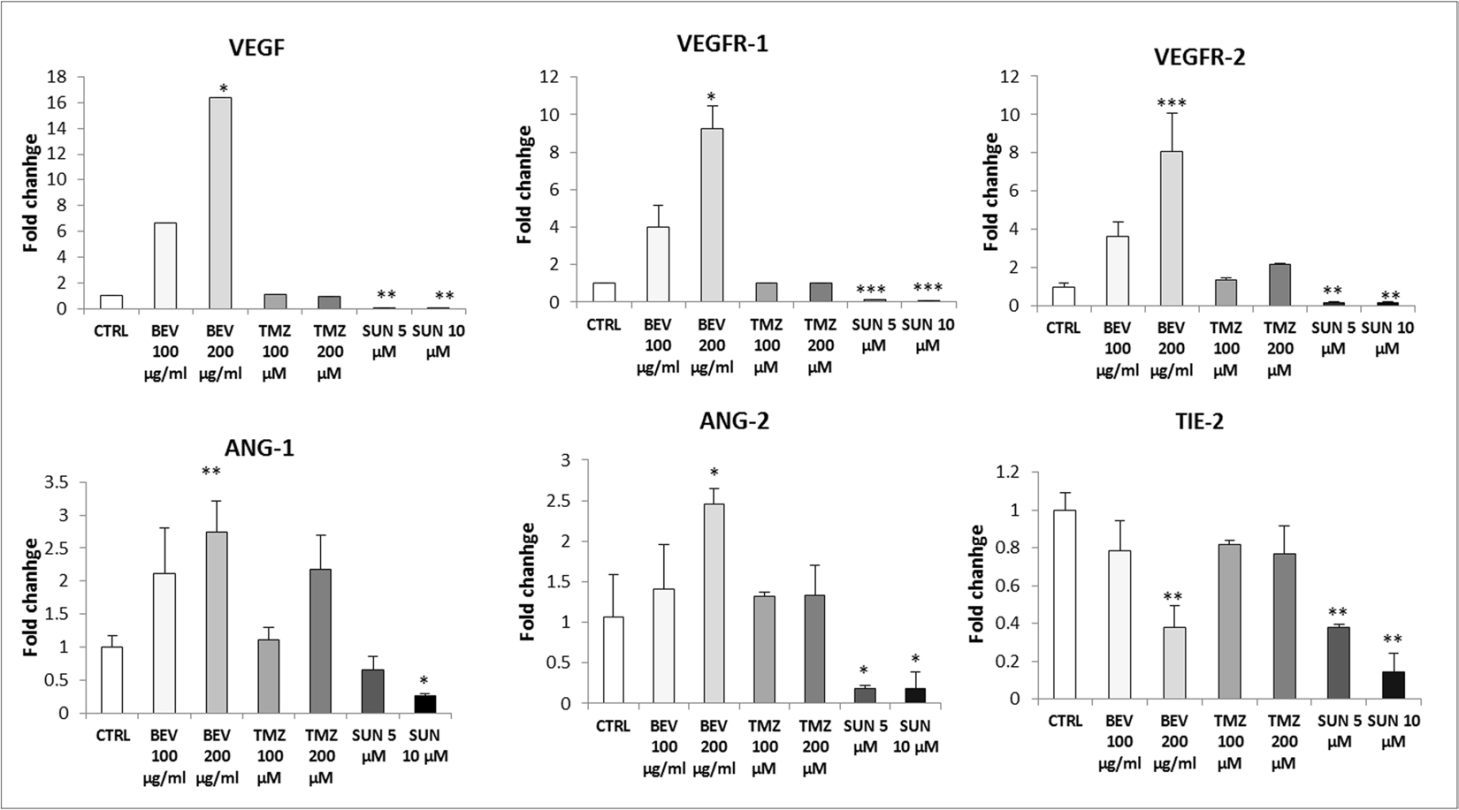
Gene expression profile of angiogenic markers on GBM-ECs after BEV, TMZ and SUN administration. qRT-PCR was performed on GBM-ECs cultured in basal condition (CTRL) or in combination with BEV 100 µg/mL and 200 µg/mL, TMZ 100 µM and 200 µM, SUN 5 µM and 10 µM for 48 h. Data were calculated using the 2^−^ΔΔCt method, normalized to 18S expression, and results are presented as a fold change relative to untreated control (CTRL). Primer sequences are listed in the Supplementary Table 2. Data are the mean of ±SD of three independent experiments. *BEV, TMZ, SUN vs CTRL. *P < 0.05, **P < 0.01, ***P < 0.001.

qRT-PCR revealed an interesting mRNA expression profile in response to 48 h of pharmacologic treatment (Fig. 5). Tumor suppressor p53 mRNA expression suffered a significant decrease after treatment with BEV and TMZ, in a dose-dependent manner. In particular, BEV showed a more effective action, especially at the higher concentration, causing a downregulation of 3-and 7-fold at 100 µg/ml and 200 µg/ml, respectively, compared to control (CTRL). A similar but less marked trend was observed after treatment with TMZ. On the contrary, p53 was up-regulated after SUN administration both at 5 and 10 µM. RAS, ERK-1, PI3K and AKT mRNA expression was not affected by BEV and TMZ, at the two administered concentrations, whereas interestingly, SUN significantly decreased their levels of about 5-fold. Regarding apoptosis regulators, BCL-2 and BAX mRNA expression underwent a significant increase after drug treatment with BEV, TMZ and SUN. Interestingly the ratio BCL-2/BAX mRNA expression was in favour of the anti-apoptotic BCL-2 for BEV and TMZ, and in favour of the pro-apoptotic BAX for SUN. Moreover, qRT-PCR was performed to evaluate the effect of drug administration on gene expression of angiogenic factors (Fig. 6). Results showed a significant overexpression of VEGF, VEGFR-1 and VEGFR-2 after treatment with BEV. An opposite effect was observed after treatment with SUN, which caused a drastic downregulation at both concentration. Regarding Ang-2 and VWF, a similar but less strong trend was observed. On the contrary, Tie-2 mRNA expression was decreased after drug treatment with BEV and SUN. All gene expression alteration followed a dose-dependent trend. Interestingly, administration of TMZ did not affect mRNA expression levels of each investigated marker.

### T98G cells viability after TMZ administration is affected by ECs conditioned media

**Figure 7:**
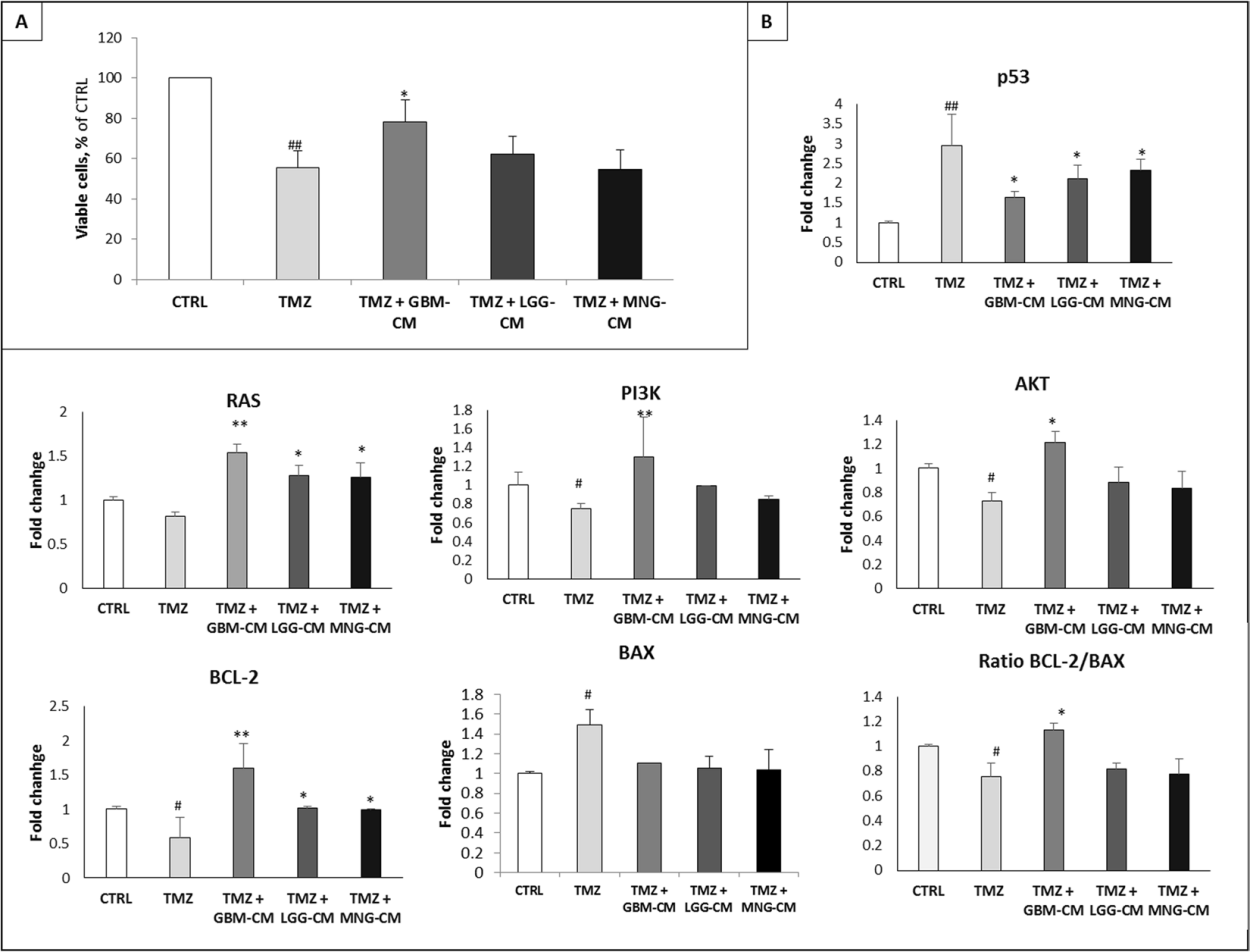
Effect of EC-conditioned media (CM) on T98G response to TMZ administration. (**A**) MTT assay was performed on T98G cultured in basal condition (CTRL), with TMZ 200 µM or in combination of TMZ and GBM-EC, LGG-EC and MNG-EC conditioned media (CM), at 24 h and 48 h after treatment. Data are presented as a percentage of viable cell relative to untreated control (CTRL). (**B**) Gene expression profile of proliferative and apoptotic markers on T98G in the same conditions listed above. Data were calculated using the 2^−^ΔΔCt method, normalized to 18S expression, and results are presented as a fold change relative to untreated control (CTRL). ^#^TMZ vs BM, *TMZ + GMB-CM,LGG-CM,MNG-CM vs TMZ. *^,#^P < 0.05, **^,##^P < 0.01, ***P < 0.001.

To evaluate if ECs from GBM, LGG and MNG may influence drug efficacy, co-colture experiments were performed. In particular, T98G cells were cultured with GBM-EC, LGG-EC and MNG-EC conditioned media (-CM). In this regard, MTT assay revealed that TMZ 200 µM, after 48 h, decreased cell viability of about 40%, suggesting a sensitive response to chemotherapy(Fig. 7A). Noteworthy, combined treatment of T98G cells with TMZ + GBM-CM, significantly increased cell viability of about 25% compared to TMZ alone. Notably, this effect was not observed following combined treatment with TMZ + LGG-CM and TMZ + MNG-CM.

### GBM-CM induces resistance to TMZ in T98G cells

qRT-PCR was performed to detect changes in mRNA expression of proliferation and apoptotic regulators on T98G cells, treated with TMZ alone or in combination with GBM-, LGG- and MNG-CM (Fig. 7B). Our results showed that T98G were sensitive to TMZ treatment, but its effect was suppressed when GBM-CM was added to the culture. In detail, mRNA expression of tumor suppressor p53 increased of about 3-fold after TMZ treatment, and its expression decreased significantly when T98G were treated with TMZ + GBM-CM. A similar but not so strong effect, was observed when T98G cells were treated with TMZ + LGG-CM and TMZ + MNG-CM. mRNA expression of cell survival and proliferation markers as RAS, AKT and PI3K was decreased of about 50% after TMZ administration and, also in this case, the effect of TMZ was inhibited by GBM-CM. Regarding apoptosis regulators BCL-2 and BAX, despite TMZ was able to decrease mRNA expression of the anti-apoptotic BCL-2 and to increase mRNA expression of the pro-apoptotic BAX, GBM-CM had the great effect to antagonize its action, inducing the opposite outcome.

## Discussion

Malignant gliomas are highly infiltrative tumors and this characteristic is the main factor for the inevitable tumor recurrence and short survival after aggressive therapies. The “angiogenic switch” responsible for the formation of new blood vessels, leads to an overproduction of pro-angiogenic factors, resulting in uncontrolled proliferation, infiltration and progression of tumor mass^12^. Heterogeneous vascular compartment has been described in various tumor types at different tumor stages, and even within a single tumor stage^13^. In addition to differences in vascular morphology, the endothelial fenestration pattern and gene expression profile of the respective vasculatures are often variable and affect differently therapeutic efficacy of antiangiogenic drugs with various molecular targets^14^. Our study was focused on the comparison of angiogenic properties of different grades of brain tumors, in order to evaluate ECs functionality, pro-angiogenic factor expression, and their contribution on drug resistance/sensitivity of GBM tumor cells. Starting with the phenotypic characterization, GBM showed a higher intensity labelling for CD34 than LGG and MNG ones. CD34 expression has been demonstrated to play a crucial role in the regulation of glioma angiogenesis by promoting a new blood vessel network and driving further glioma growth. According with our results, Rahmah *et al*. founded that HGGs showed higher expression of CD34 compared to LGG, maybe correlated with an higher densities of blood vessels^15^. Our results demonstrated a significant increased positivity for endothelial marker in GBM-ECs compared to LGG-ECs and MNG-ECs^15^. VWF fibers are a peculiar feature of highly activated ECs and microvascular proliferation^16^. ECs-released VWF fibers markedly bind platelets, forming aggregates that may protect cancer cells from immune cells, or may directly influence tumor extravasation of activating-ECs factors. Several reports have described a direct interaction between the staining intensity of VWF in ECs and the different grade of brain tumors^17^. Our previous studies showed that GBM patients had supraphysiological plasma VWF antigen level that correlates with poor prognosis and shorter survival^18^. Here, the overexpression of VWF was confirmed also by mRNA expression in GBM, compared not only to normal brain, but also to LGG and MNG, confirming its correlation with tumor malignancy and its potential use as prognostic factor. Upon ECs activation, VWF is released from ECs with other proangiogenic factors, as VEGF and Ang-2. VEGF is certainly the best characterized pro-angiogenic mediator and its overexpression leads to the formation of fragile microvessels, with a disrupted structure and increased permeability^19,20^. VEGF exerts its many effects promoting ECs proliferation, migration and survival and several studies reported a strictly correlation between VEGF, microvascular density and tumor grade and, consequently, with clinical outcome and prognosis^21,22^. Together with these findings, our results showed that mRNA expression of VEGF, and its receptor VEGFR-1, was significantly increased in GBM, compared to both healthy brain, LGG, and MNG. These data suggest that angiogenesis is a key mechanism of GBM progression, but is not equally enhanced in LGG and MNG, confirming the importance of the signalling pathway VEGF/VEGFR in tumor angiogenesis. Moreover, the overexpression of HIF-1α is strictly related to glioma malignancy and several studies reported its association with poor prognosis in different types of cancer, including gliomas^23^. Also, angiopoietins (Ang) and their receptor, Tie-2, play a critical role in angiogenesis and vascular stability^24^. Ang-1, an agonist for the Tie-2 receptor, promotes interaction between eECs and peri-EC support cells to stabilize vessels, favouring structural integrity and maturation of blood vessels^25^. On the contrary, Ang-2 is an antagonist for the Tie-2 receptor that inhibits competitively binding of Ang-1, triggering endothelium activation and vascular destabilization^25^. Indeed, Ang-2 acts as a proangiogenic agent, promoting establishment of disorganized and defenestrated blood vessels with increased permeability and loss of integrity. Interestingly, in our tumor samples, Ang-2 was overexpressed in GBM compared to HB, LGG and MNG and, on the contrary, Ang-1 was significantly downregulated in GBM respect to HB, LGG and MNG, highlighting a marked vascular instability in GBM. These results were further confirmed by our blood brain barrier (BBB) model, in which GBM-ECs were not able to form a closed and functional barrier, allowing the passage of a great quantity of dextran in a time-dependent manner. This increased permeability may reflect the impairment of BBB. The leakage of BBB allows plasma proteins extravasation in extravascular space, leading to alteration of extracellular matrix and promotion of angiogenesis and it is an important parameter for drug therapy, because it might indicate lower drug availability in the tumor region^26^. The increased permeability of GBM-ECs monolayer could be due to the lack of functional adherent and tight junction. This hypothesis may be supported by the lower fluorescence intensity of VE-Cadherin labelling in GBM-ECs compared to LGG-ECs and MNG-ECs. The VE-Cadherin is a tight junction protein that plays an important role in the integrity of the BBB. Therefore, the reduced expression of junctional protein may contribute to leakiness in tumor blood vessels. The loss of VE-Cadherin expression in gliomas was recently confirmed by other investigators. In fact, it has been demonstrated that in GBMs, tumor microvessels lose the expression of several tight junction proteins^27^, as VE-Cadherin^28^. A further strengthening of our results arise from tube-like structures formation. GBM-ECs were able to form a complex network, characterized by cords, junctions and meshes, better organized and in a greater number respect to LGG-ECs and MNG-ECs, which, in the same time frame, did not succeed to form an *in vitro* vascular architecture. These results demonstrate, once again, that abnormal vascular formation is a process strongly promoted in GBM respect to LGG and MNG, and this probably correlates with the prognosis. Moreover, phenotypic analyses revealed that GBM-ECs were highly positive to CD105, endoglin, a transmembrane glycoprotein expressed on activated vascular ECs, which acts as accessory protein of the transforming growth factor receptor^29^. In several cancers, endoglin is found on peritumoral and intratumoral vessels and its overexpression was observed primarily in malignant lesions, correlating positively with patient outcome and survival rates^29^. Another marker that we found significantly positive in GBM compared to LGG and MNG, was CD90, a highly glycosylated protein belonging to the immunoglobulin superfamily, widely characterized in various types of cells in normal tissues, including neurons and fibroblasts. Recently, CD90 has become an attractive molecule for cancer research because it is now recognized as marker for cancer stem cells in malignant tumors as hepatocellular carcinomas^30^ and GBM^31^. On the other hand, CD90 is expressed on activated ECs, working as marker for tumorigenesis and angiogenesis^32^. Anyway, the high percentage of LGG-ECs positive to CD105 and CD90 may reflect the propensity of LGGs to undergo malignant transformation, developing in secondary GBMs. Another major aspect of GBM biology that contributes to its poor prognosis is the presence of a stem cell component with self-renewing capacity and differentiation ability, responsible for recurrence events. Moreover, a high percentage of GBM-ECs (about 70%), showed positivity to CD56, or NCAM, a cell-surface glycoprotein widely characterized in CNS, when it regulates intercellular adhesion and cell migration. Previous studies evidenced a positive association between NCAM expression and tumor grade, and in particular between NCAM and cancer aggressiveness^33^. This hypothesis agrees with our evidence that NCAM expression was significantly up-regulated in GBM, but not in LGG and MNG.

As mentioned above, our isolation and characterization platform of endothelial compartment of brain tumor was developed to test different drug efficacy. To this aim, in this study, we started evaluating the relationship between *in vitro* drug response of primary brain tumor ECs and their expression of selected biomarkers putatively associated with drug resistance. The cellular basis of drug resistance in malignant gliomas and other tumors seems to be associated with various mechanisms, including those that prevent drugs from reaching their target in active form, enhanced DNA repair, or disruption of the apoptotic response to DNA damage^34^. After surgical resection, the standard of care for patients with newly diagnosed GBM is concurrent radiotherapy and TMZ. TMZ is a DNA alkylating agent known to induce cell cycle arrest at G2/M and to eventually lead to apoptosis, used to treat GBM and astrocytomas^35^ The development of resistance towards cytostatic drugs like the first line drug TMZ is a problem for GBM treatment, and relapses of the disease are really common^36^. In the last few years, the anti-angiogenesis drug Avastin® (Bevacizumab, BEV), which targets VEGF, has become one of the most promising cancer drug, also in GBM^9^. Anti-angiogenic therapy may lead to vascular normalization and facilitate conventional cytotoxic chemotherapy. For these premises, we decided to test the action of TMZ and BEV on our primary isolated GBM-ECs. Our data suggested that the effects of BEV and TMZ were transient and the mechanisms of their failure were complex and multifactorial. In this regard, treatments of GBM-ECs with two different concentrations, at two time-points, revealed a time- and dose-dependent response. Indeed, both viability and tube-like structures formation suggested an inhibitory drug effect at 24 h, which was not confirmed at 48 h, when GBM-ECs recovered their endothelial functionality. Surprisingly, treatment with a novel tirosin kinases inhibitor, Sunitinib (SUN) reported a different response pattern, although also time- and dose-dependent. In fact, the effect of SUN on viability and tube formation increased overtime, without any significant functional recovery following prolonged treatment. Indeed, gene expression analysis of GBM-ECs after pharmacological treatment, revealed that tumor suppressor p53 was down-regulation by BEV and TMZ, and up-regulated by SUN. p53, the “guardian of the genome,” is certainly one of the most widely studied protein in human glioma. Activation of p53 triggers different cellular programs such as cell cycle arrest, apoptosis, differentiation, DNA repair, autophagy, and senescence through complex network and signalling pathways^37^. The activation of p53 in the cells leads to either DNA repair and recovery or apoptosis^38^ and p53 has also been shown to have an effect on apoptosis by regulating BCL-2 and BAX expression. It has been suggested that the ratio of BCL-2 to BAX may determine cell fate, survival or death, when exposed to apoptotic stimuli such as pharmacological treatment^39^. In line with these evidence, our results showed that the ratio BCL-2/BAX was in favour of BCL-2 following BEV and TMZ administration. Interestingly, SUN switched the ratio in favour of BAX, suggesting its pro-apoptotic effect in our GBM-ECs. Overall, our data suggest a resistance response pattern of GBM-ECs towards BEV and TMZ and, on the contrary, a sensitivity pattern towards SUN. On the other hand, gene expression of angiogenesis modulators revealed an opposite action of BEV versus SUN. Indeed, VEGF, VEGFR1-2 and Ang-2 were upregulated after BEV treatment, but significantly downregulated following SUN administration. This effect may be attributed to a compensatory self-regulating mechanism that govern aberrant new vessel formation and their relative instability^40^. One possible explanation is that GBM-ECs activate collateral and different signalling pathways in order to overcome angiogenesis inhibition caused by BEV. It seems that other vascular factors compensate for the function of VEGF and that therefore the angiogenesis cannot be completely inhibited by BEV, weakening its therapeutic efficiency^41,42^. On the contrary, acting as a multi-tyrosine kinase inhibitor, we demonstrated that SUN acted as an anti-angiogenic multi-target treatment, affecting more effectively ECs functionality and pro-angiogenic marker expression, suggesting its positive effect on tumor angiogenesis. The effect of SUN could be explained by targeting of different VEGF-unrelated receptor tyrosine kinases and other non-endothelial receptors such as CD117 and the receptors for Platelet Derived Growth Factor (PDGFR), and for Glial cell line-Derived Neurotrophic Factor (RET)^43^, as well as intracellular targets, showing a multifactorial effect on different signalling pathways regulating tumor cells^44,45^. We further demonstrated that ECs not only showed a resistant behaviour to pharmacological treatments but they were able to confer resistance to T98G-sensitive cells. Interestingly, T98G cultured with conditioned media from GBM-ECs, LGG-ECs and MNG-ECs changed their response profile, turning into resistant cells. Indeed, T98G treated with TMZ in combination with GBM-CM had a percentage of viable cells higher than those treated with TMZ alone, as well as higher than those treated with TMZ in combination with LGG-CM and MNG-CM. mRNA expression level confirmed this trend, reporting the interesting action of GBM-CM in counteracting TMZ effect. Notably, the effect produced by LGG-CM and MNG-CM in conferring drug resistance to TMZ was not the same as GBM-CM, confirming the aberrant pro-angiogenic microenvironment specific of GBM. Overall, our results obtained from co-culture systems indicated that GBM-CM, but not LGG-CM and MNG-CM, induced a resistant behaviour to anticancer drug TMZ in T89G cells, suggesting that the difference in tumor microenvironment or tumor malignancy may affect tumor response to drugs^46^. In conclusion, our results allow us to develop a tumor-derived EC platform, biologically as close as possible to the *in situ* situation, as “disease in a dish”, to provide a further piece of the puzzle of the different grade-related angiogenic properties of human brain cancers. This tool has a high potential to test different anti-antiangiogenic drugs, in order to improve targeted approaches to GBM therapy.

## Methods

### Tumor samples processing and EC isolation

Tissue samples from patients who underwent surgical resection of GBM, LGG, MNG (n = 5 for each type) were at the Neurosurgery Unit of Fondazione IRCCS Ca’ Granda Ospedale Maggiore Policlinico (Milan, Italy) were included in the study (Table 1). The Institutional Review Board approved the protocol and all patients provided informed consent. All research was performed in accordance with relevant guidelines and regulations. Tumor specimens were washed in D-PBS (Euroclone, Milan, Italy) and suspended in DMEM/F12 (ThermoFisher, Waltham, Massachusetts) containing 1% penicillin/streptomycin at 4 °C, for up to 24 h after surgery. For each sample, an aliquot of tissue was frozen dry at −80 °C for successive analysis, whereas another aliquot was processed by finely mincing with surgical scalpel in D-PBS and antibiotics. After shredding, samples were enzymatically digested with 0.625 Wu/ml Liberase Blendzyme 2 (Roche, Mannheim, Germany) for 1 h at 37 °C. Cells were plated into 25 cm2-flask coated with bovine type I collagen (BD Biosciences, Milan, Italy) and cultured in endothelial proliferation medium EndoPM at 37 °C, 5%CO_2_, 5%O_2_, according to published protocol by Navone *et al*.^47^, (Supplementary Fig. 1). The media were changed 1-2 times a week and passaged at a split ratio of 1:4 every 14 days ECs (n = 5 for each tumor subtype) were used for each assay.

**Table 1:** Main characteristics of brain tumor samples included in the study.

	Tumor grade	Age	Ki67	IDH status	MGMT methylation	LOH 1p/19q
GBMs	Glioblastoma (WHO IV)	61	55%	*wt*	58%	*wt*
	Glioblastoma (WHO IV)	58	30%	*wt*	30%	*wt*
	Glioblastoma (WHO IV)	59	30%	*wt*	34%	*LOH*
	Glioblastoma (WHO IV)	63	35%	*wt*	44%	*wt*
	Glioblastoma (WHO IV)	62	35%	*wt*	40%	*wt*
LGGs	Diffuse Astrocytoma (WHO II)	56	1%	*wt*	14%	*wt*
	Diffuse Astrocytoma (WHO II)	72	30%	*wt*	4%	*wt*
	Diffuse Astrocytoma (WHO II)	62	13%	*wt*	42%	*wt*
	Oligodendroglioma (WHO II)	48	1%	*mut*	22%	*LOH*
	Oligodendroglioma (WHO II)	58	10%	*mut*	10%	*LOH*
MNGs	Meningioma (WHO I)	74	13%	*/*	/	*/*
	Meningioma (WHO I)	52	1%	*/*	/	*/*
	Meningioma (WHO I)	59	15%	*/*	/	*/*
	Meningioma (WHO I)	63	7%	*/*	/	*/*
	Meningioma (WHO I)	88	5%	*/*	/	*/*

### T98G cell culture

T98G were purchased by the Istituto Zooprofilattico Sperimentale della Lombardia e dell’ Emilia Romagna (Brescia, Italy) and cultured in DMEM High Glucose additioned with 10% Fetal Bovine Serum (FBS), 1% sodium pyruvate (Sigma), 1% glutamine and 1% penicillin/streptomycin. Cells were maintained at 37 °C, 5%CO_2_ and passaged at a split ratio of 1:2 every 5 days.

### Immunohistochemistry

GBM, LGG and MNG specimens were fixed in 4% paraformaldehyde (PFA) and 0.1 M D-PBS, embedded in paraffin and sectioned (10 µm) on a microtome. After inhibition of the endogenous peroxidases with 1% H_2_O_2_, sections were incubated in 1% BSA (Sigma Aldrich) with 0.2% Triton X-100 for 1 h, and then incubated overnight with primary antibodies anti-CD34 at 4 °C. The antibody staining was revealed using a biotinylated secondary antibody diluted with 0.1% BSA in D-PBS. For detection, the Novolink Polymer Detection System was used. Immunohistochemical data from tumour tissues were independently evaluated by two histopathologists.

### Immunofluorescence analysis

ECs (1 × 10^4^/well) were seeded into µ-Slide 8 Well, ibiTreat (Ibidi, Martinsried, Germany) collagen-coated. When cells reached the desired confluence and were fixed in paraformaldehyde 4% for 20 min at RT, washed twice with D-PBS and incubated with 0.1 M glycine to quench auto-fluorescence. Then, the coverslip was incubated with PBS + 0.25%Triton x100 to permeabilize cell membranes and then blocked in PBS + 5%BSA for 30 min at RT. Incubation with primary antibodies (AB-I) diluted in blocking buffer was performed overnight at 4 °C. The following AB-I were used: anti-VWF (ThermoFisher, Waltham, Massachusetts), anti-VEGFR-1 (ReliaTech GmbH, Wolfenbüttel, Germany), and anti-VE-Cadherin (Santa Cruz Biotechnology, Heidelberg, Germany) as specific markers for endothelial cells. To confirm EC high purity immunostaining with GFAP (1:100, BD), CD68 (1:100) and smooth muscle actin (SMA, 1:50) (both from SantaCruz Biotechnology) were performed. The following day, AB-I was removed and fluorescent secondary antibody (AB-II) labelling was then added for 45 min at RT, protected from light. After incubation with AB-II and two washes with D-PBS, DAPI (1 μg/ml, ThermoFisher) staining was performed for 3 min at RT and coverlips were mounted with ProLong Gold Antifade Mountant (ThermoFisher). Immunolabeling was acquired using an inverted DMI4 microscope equipped with DFC350xCCDcamera and LAS-X software (all from Leica Microsystems, Wetzlar, Germany).

### Flowcytometric analysis (FACS)

ECs (1 × 10^5^/tube) were resuspended in 200 μl of D-PBS and stained with FITC-conjugated anti-human CD105 and PE- conjugated anti-human CD133, anti-human CD56, anti-human CD90 (all purchased by BD Biosciences), for 20 min at RT in the dark. In order to exclude dead cells from the analysis, 7-aminoactinomycin D (7-AAD, BD) was added to each tube. Flow cytometric analysis were performed using FACScalibur flow cytometer Cell Quest software (FACS, BD Bioscience, version 8.0).

### Estimation of proliferation rate

ECs (5 × 10^4^) were seeded into 25 cm^2^ collagen-coated flask, cultured in EndoPM at 37 °C, 5%CO_2_, 5%O_2_ and passaged at a split ratio of 1:2, as they were in confluence. At each passage time point, ECs detached by TrypLE Select were stained with Trypan Blue (ThermoFisher) and counted in a Fuchs Rosenthal counting chamber, to evaluate growth rate.

### Vascular permeability assay

Transwell insert (0.4 μm pore size, Corning, Corning, USA) were coated with bovine type I collagen for 45 min at 37 °C. After complete drying, GBM-ECs, LGG-ECs and MNG-ECs (2 × 10^4^) were seeded on the insert and cultured with 150 μl and 650 μl of EndoPM in the upper chamber and in the lower chamber, respectively. ECs were cultured for 3 days at 37 °C, 5%CO_2_, 5%O_2_, until they reached the appropriate confluence, and then FITC-70 kDa dextran (Sigma Aldrich) was added to the insert (4 μl, 25 mg/ml initial concentration). Every 30 min, 10 μl of EndoPM were collected from the lower chamber, for a total of 7 samples (30 min, 1 h, 1.5 h, 2 h, 2.5 h, 3 h, 6 h). After the latest time point (6 h), each sample was diluted to 200 μl with 1 × D-PBS and 100 μl of each diluted sample were transferred in duplicate into 96-well black plates. Fluorescent content of the samples was measured at 492/520 nm absorption/emission wavelengths for FITC-dextran, with Tecan Infinite 200 PRO.

### Drug treatment

In order to evaluate the effect of drug inhibition on GBM-ECs (n = 5), cell culture treatments with Bevacizumab (BEV, Avastin®, Roche) at concentrations of 100 µg/ml and 200 µg/ml, Temozolomide (TMZ, Schering-Plough, Milano) at 100 µM and 200 µM, and Sunitinib (SUN, Sigma Aldrich) at 5 µM and 10 µM, were performed for 24 h and 48 h.

In order to evaluate the influence of ECs on drug sensitivity/resistance of GBM tumor cells, conditioned media (CM) by GBM-ECs, LGG-ECs and MNG-ECs were placed on T98G cell cultures for 48 h. Briefly, GBM-ECs, LGG-ECs and MNG-ECs (1 × 10^5^/well) were cultured into 6-well cell culture plates collagen-coated in EndoPM at 37 °C, 5%CO_2,_ 5%O_2_. After 3 days, media were collected, centrifuged 300 gx5 min to eliminate cell debris, and stored at −20 °C.

### MTT assay

3-(4,5-Dimethylthiazol-2-yl)−2,5-Diphenyltetrazolium Bromide (MTT) assay was used to assess cell viability as a function of redox potential. GBM-ECs and T98G (5 × 10^3^/well) were seeded and cultured in 96-well plate for 24 h. Then, culture media were replaced with fresh media containing the specific treatments. Tests were performed in triplicate following 24 h and 48 h of treatment. At each time point, culture media were replaced with 100 μl of fresh media + 10 µl of MTT 5 mg/ml in D-PBS. After 4 h of incubation, media were removed and cells were lysed with 100 µl of 2-propanol/formic acid (95:5, by vol) for 10 min. Then, absorbance was read at 570 nm in microplate reader.

### Tube Formation Assay

µ-Plate Angiogenesis 96 Well (Ibidi) were coated with 12.5 mg/mL Matrigel (BD Bioscience), 10 μL/well, at 4 °C. After gentle agitation to ensure complete coating, plates were incubated for 30 minutes at 37 °C to allow solidification of Matrigel. ECs (1 × 10^4^/well) from GBM, LGG and MNG were seeded in triplicate in EndoPM and incubated at 37 °C, 5%CO_2_, 5%O_2_. Cord formation was monitored with an inverted Eclipse Ti-E microscope (Nikon Instruments, Florence, Italy), equipped with a high resolution cSMOS camera (Andor Zyla, Andor Technology, Belfast, UK) and NIS_Elements 4.51 software, using differential interference contrast (DIC). After 24 h of incubation, five random images were acquired and analysed with “Angiogenesis Analyzer” plugin in Image^48^. Tube formation assay was performed following the same protocol on GBM-ECs in presence or absence of BEV(100 μg/ml and 200 μg/ml) and TMZ (100 μM and 200 μM) and SUN (5 µM and 10 µM) at 24 h and 48 h after treatments.

### Quantitative real-time PCR analysis (qRT-PCR) on tissue homogenates

Tissue specimen of GBMs, LGGs and MNGs previously stored at −80 °C, were thawed and 1 ml of TRI-Reagent Solution (Thermo Fisher Scientific) was added. Homogenization of tumor samples was performed using TissueRuptor (Qiagen). GBM-ECs and T98G cells (1 × 10^5^/well) were seeded into 6-well cell culture plates and cultured with the appropriate medium until 80% confluence was reached. Then, media were replaced with fresh media containing basal medium, as control, or additioned with treatments listed above. Treatments lasted 24 h and 48 h for ECs, and 48 h for T98G cells. Total RNA was extracted following TRI-Reagent protocol and quantified with NanoDrop 1000 Spectrophotometer (Thermo Fisher Scientific). Reverse transcriptase reaction was executed using qScript cDNA SuperMix (QuantaBio), loading 1 μg of RNA (A_260_/A_280_ > 1.8), according to manufacturer’s instructions. qRT-PCR was performed using StepOnePlus™ (Thermo Fisher Scientific) using 1 μg of cDNA, forward and reverse primers (250 nM each one) and PowerUp SYBR Green Master Mix (ThermoFisher), in a final volume of 20 μl. Primers used for qRT-PCR analysis are listed in Supplementary Table 2. Data were normalized to 18S expression, used as endogenous control. Relative gene expression was determined using the 2^−^ΔΔCt method. In qRT-PCR comparing gene expression of different tumor grade, expression was compared to cDNA of healthy brain tissue (HB), purchased by Invitrogen (ThermoFisher), whereas for GBM-ECs and T98G treated, expression was compared to untreated control (CTRL).

### Statistical analysis

All analyses were done with SPSS software (release 25.0, IBM Corp., Armonk, New York). Parameters in the three groups (GBM, LGG, and MNG) were compared and analysed by a one-way analysis of variance (ANOVA). When significant differences were detected, Bonferroni *post hoc* comparisons were made. The Kruskal-Wallis test was used to compare and analyze non-parametric data following treatments in GBM-ECs and T98G cells. Differences were considered statistically significant for p < 0.05.

## Electronic supplementary material

Supplementary Information
