# Supplementary material for: Angiogenesis in human brain tumors: screening of drug response through a patient-specific cell platform for personalized therapy
Source: Sci Rep. 2018 Jun 8;8:8748. doi: 10.1038/s41598-018-27116-7 (PMC5993734; doi:10.1038/s41598-018-27116-7)
Supplement: Supplementary file 1 — Supplementary Information [file 41598_2018_27116_MOESM1_ESM.pdf]

Characterization of angiogenesis and drug response in human brain tumor endothelial cells: another brick in the wall to develop target therapy

Laura Guarnaccia[1#], Stefania Elena Navone[1#], Elena Trombetta[2], Chiara Cordiglieri[3], Alessandro Cherubini[4], Francesco Maria Crisà[1], Paolo Rampini[1], Monica Miozzo[5], Laura Fontana[5], Manuela Caroli[1], Marco Locatelli[1], Laura Riboni[6], Rolando Campanella[1§], Giovanni Marfia[1§]*.

Co-first authors

§ *These authors equally contributed to the study*

1. Laboratory of Experimental Neurosurgery and Cell Therapy, Neurosurgery Unit, Fondazione IRCCS Ca' Granda Ospedale Maggiore Policlinico, University of Milan, Italy.
2. Flow Cytometry Service, Laboratory of Clinical Chemistry and Microbiology, Fondazione IRCCS Ca' Granda Ospedale Maggiore Policlinico Milan, Italy.
3. Istituto di Genetica Molecolare "Romeo ed Enrica Invernizzi", Milan, Italy.
4. Cell Factory, Unit of Cell Therapy and Cryobiology, Fondazione IRCCS Ca' Granda Ospedale Maggiore Policlinico, Milan, Italy.
5. Division of Pathology, Department of Pathophysiology and Transplantation, University of Milan, Fondazione IRCCs Ca' Granda Ospedale Maggiore Policlinico, Milan, Italy.
6. Department of Medical Biotechnology and Translational Medicine, LITA-Segrate, University of Milan, Milan, Italy.

Corresponding author:

Giovanni Marfia

Tel.: +39 0255024268

Fax: +39 0255038821

Email: giovanni.marfia@unimi.it, giovanni.marfia@policlinico.mi.it

Laboratory of Experimental Neurosurgery and Cell Therapy, Neurosurgery Unit, Fondazione IRCCS Ca' Granda Ospedale Maggiore Policlinico, University of Milan, Milan, Italy

via Francesco Sforza 35, 20122, Milan, Italy.

Supplementary Fig. 1. Flow chart for isolation of brain tumor-derived ECs.

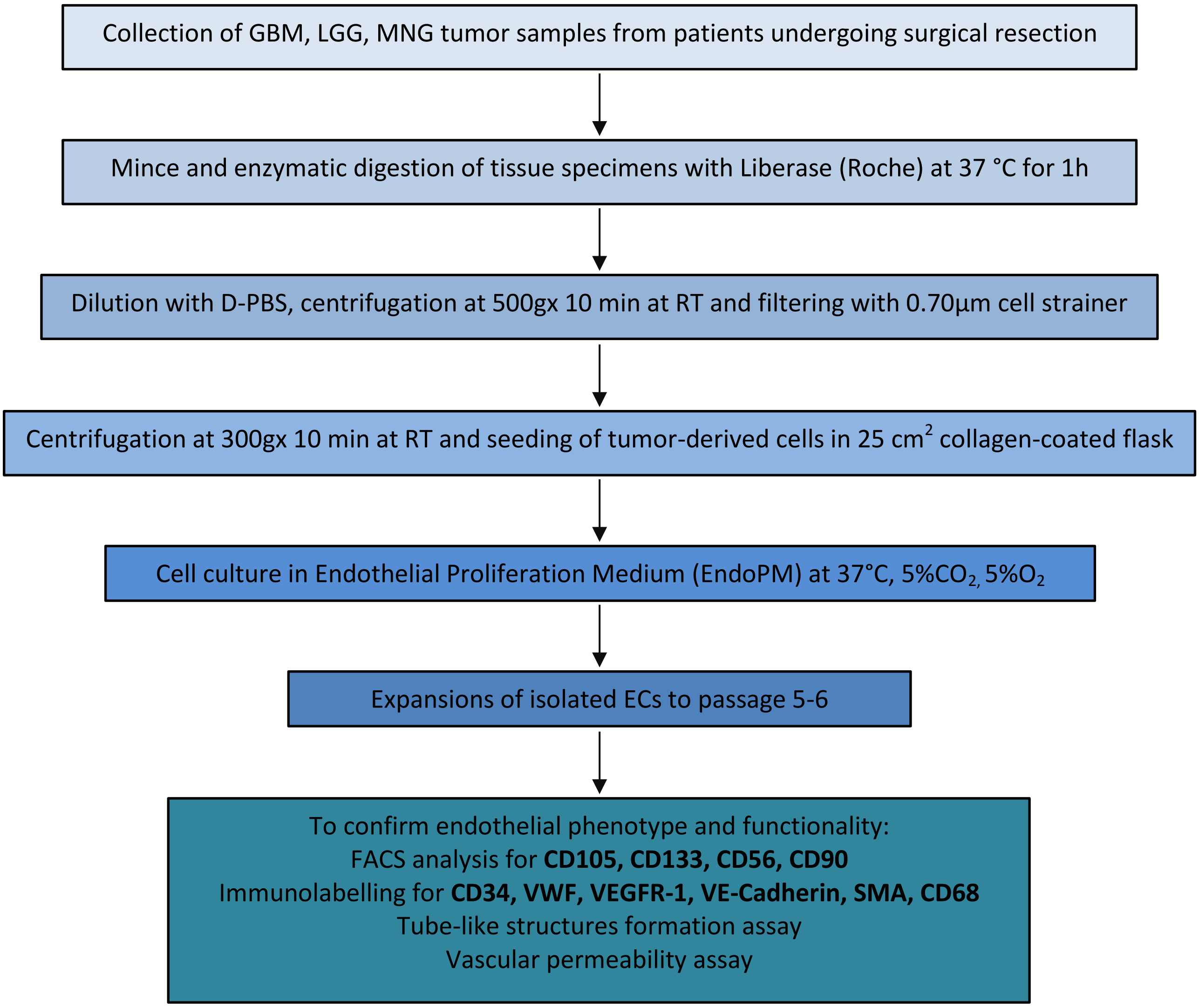

Supplementary Table 1. Primer sequences for qRT-PCR.

Gene	Forward Primer (5'- 3')	Reverse Primer (5'- 3')	T_m
**18S**	ACTTTCGATGGTAGTCGCCGT	CCTTGGATGTGGTAGCCGTTT	61 °C
**VEGF**	ACTGCCATCCAATCGAGACC	CGGCCGCGGTGTGTCTA	60 °C
**VEGFR-1**	GCAAAGCCACCAACCAGAAG	ACGTTCAGATGGTGGCCAAT	59 °C
**VEGFR-2**	GAGGGGAACTGAAGACAG	GGCCAAGAGGCTTACCTAGC	60 °C
**VWF**	ACACCTGCATTTGCCGAAAC	ATGCGGAGGTCACCTTTCAG	59 °C
**ANG-1**	GGGCACACTCATGCATTCCT	GGTTGCACATCCAAGCCAAG	60 °C
**ANG-2**	CCTGTTGAACCAAACAGCGG	GTGGGGTCCTTAGCTGAGTT	60 °C
**TIE-2**	GGAAGGTGCCATGGACTTGA	GTCATCCTCTGTATGCCTTGCT	61 °C
**NCAM**	GCAGCGAAGAAAAGACTCTGG	ATCCTCTCCCATCTGCCCTT	60 °C
**FGF-2**	TCCACCTATAATTGGTCAAAGTGGT	CATCAGTTACCAGCTCCCCC	69 °C
**p53**	AGGCCTTGGAACTCAAGGAT	CCCTTTTTGGACTTCAGGTG	58 °C
**RAS**	AGCAGGTGGTCATTGATGGG	CCGTTTGATCTGCTCCCTGT	60 °C
**ERK-1**	ACTCCAAAGCCCTTGACCTG	CTTCAGCCGCTCCTTAGGTA	60 °C
**PI3K**	GCTCCTAGCAGAAGCCTATG	TCTGGTCCTCCCGGTACA	60 °C
**AKT**	TCTATGGCGCTGAGATTGTG	CTTAATGTGCCCGTCCTTGT	58 °C
**BAX**	AGCAAACTGGTGCTCAAGG	TCTTGGATCCAGCCCAAC	57 °C
**BCL-2**	AGTACCTGAACCGGCACCT	GCCGTACAGTTCCACAAAGG	58 °C

Supplementary Table 2. Antigen expression of brain tumor tissues and ECs-derived in immunohistochemistry and immunofluorescence

	GBM tissue/EC colture	LGG tissue/EC colture	MNG tissue/EC colture
**CD34**	+++	+	+/-
**VWF**	+++	++	+
**VEGFR-1**	+++	++	+
**VE-Cadherin**	+	++	++
**GFAP**	+	+	-
**SMA**	-	-	-
**CD68**	-	-	-
